# Autonomic and Somatic Nerve Functions in Type 2 Diabetes Mellitus Patients: Electrophysiological Aspects

**DOI:** 10.3390/diagnostics11112005

**Published:** 2021-10-28

**Authors:** Anca Motataianu, Laura Barcutean, Zoltan Bajko, Adina Stoian, Smaranda Maier, Septimiu Voidazan, Rodica Balasa

**Affiliations:** 1 Neurology 1 Clinic, Emergency Clinical County Hospital Mureș, Gh. Marinescu Str., No. 50, 540136 Târgu Mureș, Romania; motataianuanca@gmail.com (A.M.); bzoltan2003@yahoo.com (Z.B.); cretadina@yahoo.com (A.S.); maier_smaranda@yahoo.com (S.M.); rodica.balasa@umfst.ro (R.B.); 2 Department of Neurology, University of Medicine, Pharmacy, Science and Technology “George Emil Palade” of Târgu Mureș, Gh. Marinescu Str., No. 38, 540139 Târgu Mureș, Romania; 3 Department of Pathophysiology, University of Medicine, Pharmacy, Science and Technology “George Emil Palade” of Târgu Mureș, Gh. Marinescu Str., No. 38, 540139 Târgu Mureș, Romania; 4 Department of Epidemiology, University of Medicine, Pharmacy, Science and Technology “George Emil Palade” of Târgu Mureș, Gh. Marinescu Str., No. 38, 540139 Târgu Mureș, Romania; septi_26_07@yahoo.com

**Keywords:** diabetic neuropathy, diabetes mellitus, heart rate variability, nerve conduction studies

## Abstract

Objectives: To investigate the relationship between neurophysiological sensory and motor nerve function parameters, assessed by nerve conduction studies (NCS) with parasympathetic autonomic function and by heart rate variability (HRV) tests in patients with type 2 diabetes mellitus (T2DM). Material and Methods: A total of 161 T2DM patients underwent NCS. Cardiac autonomic response was assessed by HRV tests to deep breathing (HRV DB), to Valsalva manoeuvre, and during postural change from lying to standing. Results: The amplitude of motor response in the median nerve, tibial nerve, and peroneal nerve was associated with reduced HRV DB (*p* = 0.0001). The amplitude of motor response in the median nerve, tibial nerve, and peroneal nerve was associated with reduced HRV Valsalva (*p* = 0.0001). The correlation between the amplitude of response in all sensory nerves (sural, median, and ulnar) and HRV DB was statistically significant (*p* = 0.0001). Conclusion: The results indicate that there is a correlation in T2DM patients between the damage of small myelinated and unmyelinated nerve fibres from cardiac autonomic nerves, assessed by HRV tests and damage of large motor and sensory fibres, assessed by NCS. Based on the above results, a combination of NCS and HRV tests should be considered in the neurophysiological approach to diabetic neuropathy.

## 1. Introduction

Disorders of the peripheral nervous system in diabetes are common and complex conditions. Disorders may involve sensory fibres, motor fibres, small myelinated fibres, and autonomic fibres, producing a heterogeneous group of clinical manifestations. The most common forms of diabetic neuropathies in clinical practice are distal symmetric polyneuropathy (DSPN), which affects somatic sensory fibres in the early stages, followed by motor fibre involvement from peripheral nerves and autonomic neuropathies [1,2].

Up to 50% of diabetic peripheral neuropathies can be clinically asymptomatic. Clinical electrodiagnostic studies, assessed by nerve conduction studies (NCS), have become an indispensable tool for the discovery of peripheral nerve abnormalities. NCS is a noninvasive, accurate, sensitive, and reliable method that provides objective and quantitative data concerning somatic motor and sensory nerve fibre function. NCS can also detect subclinical abnormalities [1,3].

Cardiac autonomic neuropathy (CAN) is the most important form of autonomic neuropathy and often is an underdiagnosed complication in diabetes. CAN and sympathetic/parasympathetic imbalance are important causes of morbidity and mortality in diabetes due to the high risk of cardiac arrhythmias and sudden death, likely related to silent myocardial infarction [1,4,5].

Decreased heart rate variability (HRV) has been accepted as an early biomarker of CAN in diabetes. Cardiac autonomic dysfunction, together with somatic neuropathy, has become a powerful predictor of mortality risk in patients with diabetes. This implies that evaluating nerve conduction parameters and HRV could be lifesaving. HRV evaluation is very important for patient risk stratification and therapeutic interventions. This assessment has a greater significance now, as we have medications that can prevent the progression of autonomic nervous system dysfunction [6,7,8,9,10].

Despite their common occurrence, DSPN and CAN are not as reliably diagnosed as would be desirable. Clinical diagnosis from signs and symptoms is excessively variable, so specific approaches including NCS and autonomic tests are necessary [8]. Early cardiac autonomic dysfunction can only be detected by reduced heart rate variability (HRV) to deep breathing (HRV DB). CAN should be diagnosed per the Toronto Consensus Panel and ADA Position Statement, using standardised cardiac autonomic reflex tests that assess changes in the heart rate response (changes in R-R interval on electrocardiogram recordings) to deep breathing, Valsalva, and standing position [1,10,11,12,13].

In the present study, we looked to analyse the spectrum of electrophysiological parameters of somatic and parasympathetic autonomic nerve function in a large sample of T2DM patients, early in the time course of the disease and without apparent organ dysfunction, in order to correlate cardiac autonomic parameters with somatic nerve function parameters.

## 2. Materials and Methods

### 2.1. Subjects

One hundred and sixty-one T2DM patients were included in this cross-sectional study. Study patients meet the American Diabetes Association criteria for T2DM. Patients were excluded based on the following criteria: secondary causes of diabetes, hypo- or hyperglycaemic episodes 24 h prior to examination, advanced diabetic retinopathy, vitamin B12 deficiency, recent history of infections, diabetic ketoacidosis, alcohol consumption, other causes of neuropathy, cardiac diseases such as heart blocks, arrhythmias, marked tachycardia (over 105 beats per minute), coronary artery disease and cardiomyopathy, any acute conditions, malignancies, thyroid disease, severe systemic involvement (pulmonary, kidney or cardiac insufficiency), antiarrhythmic medication, antidepressants, antihistaminic agents, sympathomimetic cough agents or chemotherapeutic agents that can affect the autonomic and somatic nervous system, as well as other medications that could interfere with the cardiac rhythm (including antidiabetics such as sulfonylureas) and other causes of neuropathy [4]. The study protocol was approved by the Research Ethics Board of the University of Medicine and Pharmacy Targu Mures, No. 20/1.06.2012, and all subjects gave their written informed consent.

### 2.2. Electrophysiological Studies in Somatic Nerves

For all patients, peripheral somatic nerve function was assessed using nerve conduction studies (NCS) via an electrodiagnostic protocol as recommended by the American Association of Electrodiagnostic Medicine [14]. For each patient, NCS were performed bilaterally on the median, ulnar, peroneal, tibial, and sural nerves according to standard techniques [15]. For the statistical analysis, the mean of two matching NCS values of the same nerve was used. NCS were performed using a four-channel electromyography (EMG) apparatus (Nihon Kohden, Neuropack MEB-9400, Tokyo, Japan) with surface electrodes. Limb temperatures were measured before NCS, and the skin temperature was more than 34.0 °C for the hands and 33.0 °C for the feet. All electrophysiological tests were performed by the same examiner. Normal electrophysiological values were also added in Table 2 for ease of interpretation, based on standardised values [16].

The following were evaluated in the motor nerves: distal motor latencies (DML), motor nerve conduction velocities (MNCV), and compound muscle action potential amplitudes (CMAPA). The following sites are included for the analysis of evoked CMAPs: ulnar nerve (wrist and below the elbow), median nerve (wrist and elbow), tibial nerve (ankle and popliteal fossa) and peroneal nerve (ankle and below fibular neck). The amplitudes of the CMAP in motor nerves were measured from baseline to peak.

In sensory nerves, the following electrophysiological parameters were evaluated: sensory nerve action potential amplitudes (SNAPA), sensory nerve conduction velocities (SNCV), and distal sensory latencies (DSL). The amplitudes of the SNAP were measured from baseline to negative peak. The motor and sensory nerve latencies were measured at the onset of the initial deflection from baseline. The conduction velocities in both sensory and motor nerves were automatically calculated.

### 2.3. Cardiac Parasympathetic Autonomic Testing

Patients avoided any physical exercise in the 24 h preceding the cardiovascular testing and avoided smoking, eating, or coffee consumption for at least two hours prior to autonomic testing. All antidiabetic and other medications were administered at the end of the examination.

Three noninvasive autonomic tests were performed early in the morning, according to Ewing′s methodology, in order to evaluate the parasympathetic cardiac autonomic function [17,18]. Heart rate variability (HRV) to deep breathing with a rate of six regular breaths per minute, to the Valsalva manoeuvre, and to a postural change from lying to standing was used in order to evaluate the parasympathetic cardiac autonomic function. Electrocardiographic recordings of R-R intervals were used to assess the HRV, using an ELI 350 electrocardiograph system (Mortara Instrument Inc., Milwaukee, WI, USA). The HRV to the Valsalva manoeuvre (HRV Valsalva) was calculated using the Valsalva ratio between the longest R-R interval to the shortest R-R interval, assessed during forced exhalation into a mouthpiece of a manometer to 40 mmHg for 15 s. Exhalation to inhalation (E/I) ratio was calculated by the ratio of the maximum to the minimum heart rate, recorded during 6 cycles of paced deep breaths. The HRV to postural changes was evaluated by the ratio of the longest R-R interval during beats 20–40 after standing to the shortest R-R interval during beats 5–25 after standing. HRV to deep breathing was assessed by recording the difference between the maximum and minimum heart rates (beats/minute) during six breaths per minute. These tests were performed using technique-specific normative data as previously described [18]. The test results of the deep-breathing tests were interpreted according to normal age-related values [19].

### 2.4. Statistical Analysis

The continuous variables were expressed by descriptive statistics as mean ± standard deviation (SD) or median (minimum–maximum), while the categorical variables were summarised by absolute and relative frequencies. The correlation between quantitative variables was assessed using Pearson correlation or Spearman′s rho, when appropriate. The confidence interval (CI) was set at 95%. The correlation coefficient, r, describes the strength of the relation as follows: 0.0–0.19, very weak; 0.20–0.39, weak; 0.40–0.59, moderate; 0.60–0.79, strong; and 0.80–1.0, very strong. Multivariate analysis was carried out using linear regressions. The dependent variables were HRV DB and HRV Valsalva. The level of statistical significance was set at *p* < 0.05.

Statistical analysis was carried out using the SPSS for Windows (v. 20.0, IBM Corporation, Armonk, NY, USA) and MedCalc (v. 10.3.0.0, MedCalc Software, Ostend, Belgium) software.

## 3. Results

### 3.1. General Characteristics of Study Patients

One hundred and sixty-one patients with T2DM were evaluated. The basic clinical and demographic information for subjects is shown in Table 1.

### 3.2. Correlations between Parasympathetic Function and Motor Nerve Function

The descriptive statistics of the mean NCS values are represented in Table 2.

**Figure 1:**
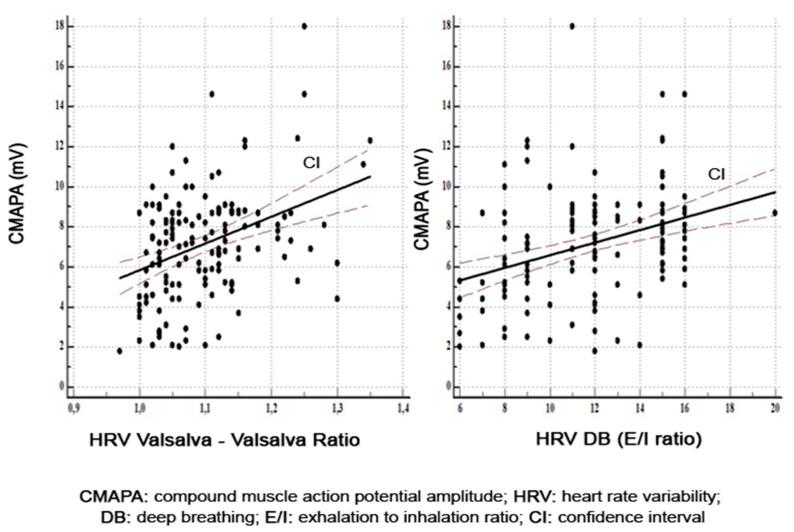
Correlations between amplitude of motor response in median nerves and HRV Valsalva and HRV DB.

A total of 2254 nerves (1288 motor nerves and 966 sensory nerves) were evaluated by NCS. A total of 483 HRV tests were performed. The correlations between the motor parameters and HRV are described in Table 3. Within the median nerve, there was a direct correlation between axonal damage and parasympathetic nerve dysfunction revealed by HRV to Valsalva (*r* = 0.37, *p* = 0.0001) and HRV DB (*r* = 0.35, *p* = 0.0001) (Figure 1).

**Figure 2:**
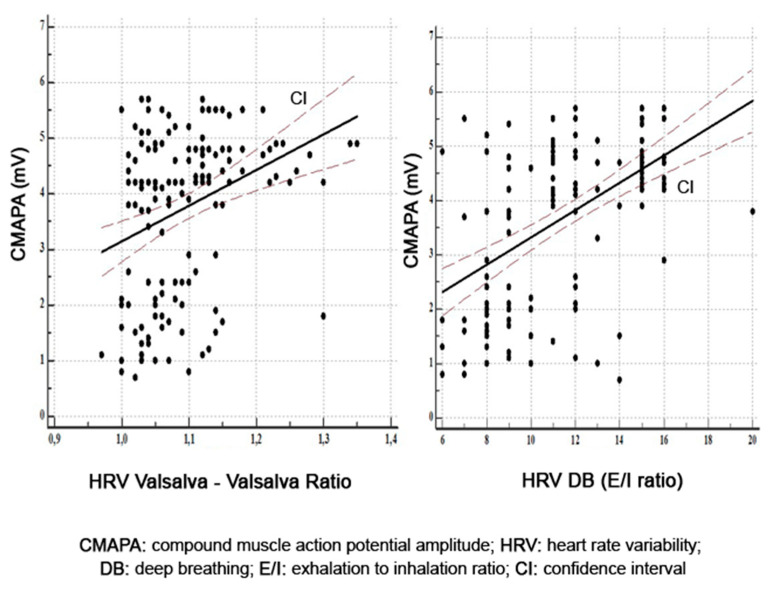
Correlations between amplitude of motor response in peroneal nerve and HRV Valsalva and HRV DB.

The univariate analysis in the peroneal nerve found the strongest significant statistic correlation between the amplitude of motor response and HRV DB (*r* = 0.52, *p* = 0.0001) and HRV Valsalva (*r* = 0.33, *p* = 0.0001) (Figure 2). Motor conduction velocity in the peroneal nerve correlated only with HRV DB (*r* = 0.38, *p* = 0.0001).

**Figure 3:**
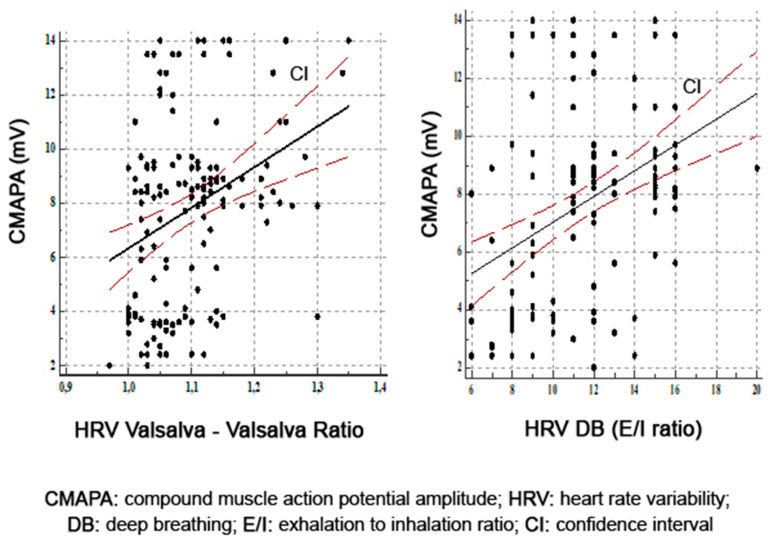
Correlations between amplitude of motor response in tibial nerves and HRV Valsalva and HRV DB.

In the tibial nerve analysis, we found a statistically significant positive correlation between amplitude of motor response and HRV DB (*r* = 0.39, *p* = 0.0001) and HRV Valsalva (*r* = 0.32, *p* = 0.0001) (Figure 3). Motor conduction velocity in the tibial nerve correlated with HRV Valsalva (*r* = 0.39, *p* = 0.0001) and with HRV DB (*r* = 0.34, *p* = 0.0001).

### 3.3. Correlations between Parasympathetic Function and Sensory Nerve Function

The correlations between the sensory parameters and HRV are described in Table 4. The amplitude of response in all sensory nerves was significantly correlated with HRV DB as follows: in the sural nerve, *r* = 0.57, *p* = 0.0001; in the median nerve, *r* = 0.46, *p* = 0.0001; and in the ulnar nerve, *r* = 0.44, *p* = 0.0001. A slight correlation was found between amplitude of response in sensory nerves and HRV Valsalva. Sensory conduction velocity in the ulnar nerve was correlated with HRV Valsalva (*r* = 0.29, *p* = 0.0002) and HRV DB (*r* = 0.29, *p* = 0.0002).

In order to evaluate the relation between the sensory–motor conduction parameters and age, diabetes duration, and HgbA1c levels, we performed the analysis of the strength and direction of the association by using Spearman′s correlation. The results are summarised in Table 5. We found statistically significant correlations between the sensory–motor conduction parameters and diabetes duration (*p* < 0.05). The strongest correlations for diabetes durations were for: motor median CMAPA (*r* = −0.51, *p* < 0.0001), motor ulnar CMAPA (*r* = −0.53, *p* < 0.0001), motor peroneal CMAPA (*r* = −0.61), *p* < 0.0001), motor tibial CMAPA (*r* = −0.55, *p* < 0.0001), sensory median SNAPA (*r* = −0.59, *p* < 0.0001), sensory ulnar SNAPA (*r* = −0.60, *p* < 0.0001), and sensory sural (*r* = −0.61, *p* < 0.0001).

### 3.4. Multivariate Analysis between Parasympathetic Function and Sensory–Motor Nerve Function

To further explore the relationship between HRV tests and sensory–motor conduction parameters, we performed a multivariate regression analysis of HRV DB and HRV Valsalva (as dependent variables), using variables from Table 1 and Table 3. In the multivariate analysis, the only variables independently associated with reduced HRV Valsalva were the amplitude in the motor median (β = 0.38, *p* = 0.01), motor ulnar (β = 0.30, *p* = 0.02), and sural nerve (β = 0.23, *p* = 0.04) (Table 6).

In the multivariate analysis, the only variables independently associated with reduced HRV DB were the amplitude in the peroneal nerve (β = 0.62, *p* = 0.01), conduction velocity in the peroneal nerve (β = 0.29, *p* = 0.01), and amplitude in the sural nerve (β = 0.66, *p* = 0.0001) (Table 7).

## 4. Discussion

The involvement of the somatic and the autonomic nervous system is most likely one of the most common complications of diabetes.

We assessed the implication of the autonomic nervous system by Ewing tests (HRV DB, HRV OC, and HRV Valsalva) and the peripheral nervous function (somatic nervous system) by using NCS. Both autonomic and somatic nerve fibres are components of the peripheral nervous system [17,20]. Our results demonstrate that there is a convergent deterioration of somatic and parasympathetic nerve function in clinically mild diabetes. The axonal damage in the sural nerve was the most important somatic neurophysiological parameter correlated with parasympathetic nerve function deterioration.

Specific for the vagus nerve is a complex mixture of nervous fibres The unmyelinated nerve fibres (A δ, thin unmyelinated C fibres) originate from the dorsal nucleus of the vagus, while the myelinated nerve fibres (Aα and Aβ) originate from the nucleus ambiguous [21,22]. From an evolutionary point of view, the unmyelinated nerve fibres appear to be the oldest, and they seem to be implicated in downregulation of the HRV when faced with an acute setting, while the myelinated nerve fibres, which are phylogenetically newer, have the capacity to augment the heart rate on demand and might be involved in rather more chronic conditions. The visceral sensory component is responsible for the modulation of the heart rate when faced with autonomic dysfunction. Most likely, the myelinated component is the dominant one, which is why we have a concordance between somatic myelinated and autonomic myelinated nerve dysfunction [23]. This hypothesis explains the convergent involvement of parasympathetic and somatic nerve dysfunction in T2DM [21].

Decreased HRV, an early biomarker of CAN, reflects impaired parasympathetic innervation of the heart by the vagus nerve. The damage in the vagus nerve, which is the longest autonomic nerve, is in a length-dependent model, like in the somatic peripheral nerves in DSPN. The concept of the natural history of diabetic neuropathy (DN) is that small, unmyelinated, and thinly myelinated A δ- and C-type nerve fibres are the most vulnerable, and the initial injury is in these fibres [24,25].

From a pathogenetic mechanism perspective, peripheral neuropathy and autonomic neuropathy in diabetes share similar multifactorial pathophysiological pathways, including polyol pathway, glycation, reactive oxygen species, and altered protein kinase C activity. These lead to damage in both somatic (large myelinated fibres) and cardiac autonomic fibres (unmyelinated and myelinated vagal fibres) [26,27]. Damage of small nerve fibres is considered the earliest alteration in the course of diabetic neuropathy, and small-fibre neuropathy represents a significant component of DSPN. Therefore, assessment of functional abnormalities of small nerve fibres may enable timely diagnosis [25,28].

In diabetes, there is symmetrical diffuse and fibre-length-dependent damage of somatic and autonomic nerves so that the longest nerves are most likely to be affected by the “die back” mechanism. The DN is a progressive distal axonopathy, so the NCS revealed abnormalities with reduced amplitude of motor and sensory response [29]. As demonstrated in our study, the increased duration of T2DM revealed a reduced amplitude of sensory and motor nerves (axonopathy) and a decreased motor and sensory nerve velocity due to the loss of fast-conducting axons. Zhang et al. [30] showed that sensory nerves were affected to a greater extent than motor nerves in DN, and the sensory nerve action potential amplitude in the sural nerve was the first abnormality to appear before motor amplitude reduction in peroneal and tibial nerves. Bi et al. [31] demonstrated that amplitude of sensory nerve action potential was a more sensitive parameter than nerve conduction velocity for detecting early diabetic neuropathy. Our findings are consistent with the above-mentioned studies, the mean sensory values, SNAPA and SNCV, being more affected than the mean motor NCS values, CMAPA and MNCV.

Our study now provides more detailed information about the relationship between NCS parameters and parasympathetic function tests assessed by HRV. We found that the highest positive correlation between motor nerve function tests and parasympathetic function tests was between the amplitude of motor response in peroneal nerves and HRV DB. The highest positive correlation between a cardiac parasympathetic test and any sensory–motor fibre function tests was between the HRV DB and the SNAPA in the sural nerve. This was the most powerful correlation between an electrophysiological parameter of somatic nerve function and parasympathetic nerve function tests. The multivariate analysis confirmed that the reduction in the sensory response in the sural nerve (longest sensory nerve) is positively correlated with a reduction in HRV in Valsalva and HRV DB, which suggests a concomitant involvement of sensory-somatic and parasympathetic nerve fibres in T2DM. The differences between biophysical properties (excitability) and in ion channel functions between motor and sensory axons can subject the sensory fibres to greater metabolic disturbances; therefore, in early DN stages, the sensory axons from the peripheral nerves are initially affected [32].

Although autonomic neuropathy affects all systems of the body, the cardiovascular parasympathetic tests are simplest to measure noninvasively and easiest to quantify in order to evaluate autonomic nerve damage in DM.

In a cross-sectional study of diabetic neuropathy and the relationships among different neurophysiological tests that assess the function of large, small, and autonomic nerve fibres, a modest relationship was identified between these tests [33]. Because diabetes can impact the function of the peripheral nervous system in a multitude of ways, the use of multiple tests is required in order to evaluate all this fibre function of the complex clinical pattern of diabetic neuropathy.

The presence of CAN negatively impacts the prognosis of T2DM patients as an independent mortality risk factor. The ACCORD study enrolled more than 10,000 T2DM patients and demonstrated an all-increased risk for mortality in individuals with different degrees of CAN [34]. In terms of glycemic control, most of the patients evaluated in our study had an HbA1c higher than 8%. Numerous studies, including the ACCORD, analysed the associated risks of hypo- and hyperglycemia and demonstrated that the incidence of mortality is higher in hypoglycaemic T2DM patients due to the risk of malignant arrhythmias and sudden cardiac death [34,35,36].

Our results in T2DM are in good accordance with other studies on type 1 diabetes mellitus (T1DM). In a study on neuropathy in T1DM patients, a strong incremental association between decreased HRV DB and increasing severity of DSPN on electrophysiological examination was identified. HRV can be used as a marker of DSPN in both early and advanced stages of neuropathy [37]. Sveen et al. [38] demonstrated in a long-term follow-up study on T1DM patients that small-fibre dysfunction was more prevalent than large-fibre dysfunction after a long duration of T1DM, and small-fibre neuropathy was associated to a limited extent with traditional cardiovascular risk factors.

Töyry et al. demonstrated in a non-insulin-dependent T2DM cohort of patients that deterioration of the sympathetic and parasympathetic function values was not related to the worsening in the sensory–motor nerve function parameters. A possible explanation of this divergent result compared to our study can be explained by a different methodology of the study design: our study included only parasympathetic autonomic assessment (HR DB, HR OC, and HR Valsalva), while their study combined both sympathetic and parasympathetic autonomic nervous function test [39]. In a recent study on the differences in the spectrum of diabetic neuropathy and progression of large- and small-fibre involvement in T1DM and T2DM, Løseth et al. found that the reduction in small fibres predominated and progressed more rapidly than large fibres in patients with T2DM [40].

Others have documented that small-fibre damage precedes large-fibre damage in diabetes, and there is a need for a new diagnostic algorithm for evaluation of neuropathy in diabetes in order to incorporate small-fibre evaluation for these patients [28,41].

An opposed opinion was published by Yun-Ru et al. They demonstrated that the sural-nerve-sensitive amplitude was positively correlated with HRV DB in T2DM patients with different durations of the disease [42]. Therefore, T2DM patients with a longer disease duration had a more severe CAN and DSPN.

Our previous study results advocate the concurrent development of somatic and autonomic nerve fibre damage in T2DM based on clinical and electrophysiological studies [43]. The initial nerve fibre damage takes place in the small myelinated and unmyelinated nerve fibres, including autonomic nerves, whereas the damage to large fibres is mostly detected in advanced phases of T2DM [44].

DSPN and CAN are considered distinct clinical entities of diabetic neuropathy, distinguished by different clinical features, but they share a common pathogenesis of nerve damage [1]. Our study demonstrates that abnormalities that occurred in the autonomic nervous system are paralleled by changes in the somatic peripheral nerve fibres. To our knowledge, this is the first study that shows a plausible relationship between alteration in electrophysiological parameters of somatic and autonomic nervous function, which are commonly affected in diabetic neuropathy. The present study results can be interpreted as congruent dysfunction in somatic and parasympathetic nerve function in T2DM.

Our study has several limitations. First, we evaluated only parasympathetic function in diabetic patients without sympathetic function. Second, we did not perform subgroup analysis based on the diagnosis of CAN or DSPN. Third, this study was conducted on one hospital-based cohort. The study does not have a control group because of the complex and distressing assessment of the peripheral nerve function. The major strengths of our study are the relatively large number of patients in which extensive motor and sensory nerves were assessed by NCS and the use of three cardiovagal tests as markers of autonomic function.

It is important to assess the cardiovagal dysfunction (the most sensible test being HRV DB) because the earliest phase of CAN is frequently asymptomatic. Therefore, adding a simple test such as HRV DB in current clinical practice can alert the clinician regarding the propensity for possible additional cardiovascular complications (silent myocardial infarction, cardiac arrhythmias during physical activities, etc.).

## 5. Conclusions

Neurophysiology is an important tool in assessing the function of peripheral nerves in diabetes, providing a conclusive diagnosis of DSPN. Because the earliest sign of CAN is impaired HRV, which frequently is asymptomatic, it is important to perform cardiovagal tests in order to diagnose this complication. We propose the addition of HRV DB to the NCS in clinical practice to incorporate autonomic evaluation as a key measure in the diagnosis of all diabetic neuropathy components. The HRV is subjected to vagal control through the myelinated vagal nerve fibres, being respiratory dependent. Therefore, HRV testing is a sensible evaluation of the autonomic function. CAN underdiagnosis can be improved by education and adaptability of our resources as clinicians in order to deal with this complex diabetic neuropathy.

## Figures and Tables

**Table 1:** Demographic and clinical characteristics of the study population.

Variable	T2DM Patients
Patients number	161
Male/Female, no. (%)	76 (47.2)/85 (52.8)
Age (years)	58.1 ± 8.2
Minimum age	33
Maximum age	77
Age at diabetes diagnosis (years)	49.8 ± 9.2
Diabetes duration (years)	6 (1–37)
<5 years, no. (%)	61 (37.9)
5–10 years, no. (%)	28 (17.4)
11–15 years, no. (%)	54 (33.5)
>15 years, no. (%)	18 (11.2)
Body mass index (kg/m^2^)	30.8 ± 5.3
Systolic BP (mmHg)	143.4 ± 19.6
Diastolic BP (mmHg)	81.4 ± 9.9
Heart rate (beats/minute)	83 (51–102)
Hypertension (yes), no. (%)	106 (65.8)
Ex-smokers, no. (%)	92 (57.1)
Smokers (yes), no. (%)	69 (42.9)
<20 cigarettes/day, no. (%) >20 cigarettes/day, no. (%)	33 (47.8) 36 (52.2)
Triglycerides (mg)	178 (60–1100)
Cholesterol (mg)	228 ± 51.3
HgbA1c (%)	8.3 ± 1.4
<6.5, no. (%)	14 (8.7)
6.5–6.9, no. (%)	16 (9.9)
7–8, no. (%)	40 (24.8)
>8, no. (%)	91 (56.5)
FPG (mg)	183.9 ± 64.0
Waist-to-hip ratio (cm)	0.90 ± 0.08
Abdominal circumference (cm)	104.6 ± 12.3

HgbA1c: glycosylated haemoglobin; FPG: fasting plasma glucose.

**Table 2:** Descriptive statistics of NCS values (mean ± SD).

Nerve		Mean ± SD		Normal Values (Mean ± SD)
Motor	Median nerve	CMAPA	6.98 ± 2.60 mV	13.2 ± 5.0 mV
		MNCV	51.30 ± 3.86 m/s	56.7 ± 3.8 m/s
	Ulnar nerve	CMAPA	7.64 ± 2.46 mV	11.6 ± 2.1 mV
		MNCV	53.38 ± 3.47 m/s	61.5 ± 5 m/s
	Peroneal nerve	CMAPA	3.780 ± 1.44 mV	5.9 ± 2.6 mV
		MNCV	42.53 ± 2.48 m/s	47 ± 4.0 m/s
	Tibial nerve	CMAPA	7.81 ± 3.44 mV	11.6 ± 4.3 mV
		MNCV	43.90 ± 3.27 m/s	49.8 ± 6.0 m/s
Sensory	Median nerve	SNAPA	16.34 ± 6.76 µV	37 ± 19.0 µV
		SNCV	50.27 ± 5.37 m/s	64 ± 8.0 m/s
	Ulnar nerve	SNAPA	13.79 ± 4.61 µV	32 ± 20 µV
		SNCV	52.42 ± 2.98 m/s	55 ± 3.6 m/s
	Sural nerve	SNAPA	9.36 ± 4.52 µV	23.7 ± 3.8 µV
		SNCV	43.33 ± 2.60 m/s	43.3 ± 4.3 m/s

CMAPA: compound muscle action potential amplitude; MNCV: motor nerve conduction velocities; SNAPA: sensory nerve action potential amplitude; SNCV: sensory nerve conduction velocities.

**Table 3:** Results of correlations between electrophysiological parameters of motor nerve function and HRV tests.

NCS Parameters for Motor and Sensory Nerves		HRV OC		HRV Valsalva		HRV DB	
		*r*-Values	*p*-Values	*r*-Values	*p*-Values	*r*-Values	*p*-Values
Median motor	DML	−0.14	0.07	−0.12	0.11	−0.23	0.004
	CMAPA	0.13	0.0001	0.37	0.0001	0.35	0.0001
	MNCV	0.16	0.04	0.17	0.03	0.17	0.03
Ulnarmotor	DML	−0.22	0.005	−0.11	0.18	−0.05	0.55
	CMAPA	0.29	0.0002	0.24	0.002	0.35	0.0001
	MNCV	0.15	0.06	0.31	0.0001	0.32	0.0001
Peroneal	DML	−0.17	0.03	−0.23	0.003	−0.25	0.001
	CMAPA	0.20	0.01	0.33	0.0001	0.52	0.0001
	MNCV	0.10	0.22	0.22	0.007	0.38	0.0001
Tibial	DML	0.05	0.55	0.11	0.15	0.08	0.33
	CMAPA	0.27	0.0007	0.32	0.0001	0.39	0.0001
	MNCV	0.10	0.22	0.39	0.0001	0.34	0.0001

DML: distal motor latencies; CMAPA: compound muscle action potential amplitude; MNCV: motor nerve conduction velocities; HRV DB: heart rate variability deep breathing; HRV OC: heart rate variability orthostatic changes; *r*: correlation coefficient.

**Table 4:** Results of correlations between electrophysiological parameters of sensory nerve function and HRV tests.

NCS Parameters for Motor and Sensory Nerves		HRV OC		HRV Valsalva		HRV DB	
		*r*-Values	*p*-Values	*r*-Values	*p*-Values	*r*-Values	*p*-Values
Median sensory	DSL	−0.17	0.03	−0.17	0.03	−0.28	0.0005
	SNAPA	0.28	0.0004	0.34	0.0001	0.46	0.0001
	SNCV	0.09	0.25	0.15	0.06	0.25	0.001
Ulnar sensory	DSL	−0.03	0.63	−0.05	0.49	−0.15	0.06
	SNAPA	0.27	0.0007	0.39	0.0001	0.44	0.0001
	SNCV	0.17	0.03	0.29	0.0002	0.29	0.0002
Sural	DSL	−0.03	0.7	−0.01	0.9	−0.14	0.07
	SNAPA	0.20	0.01	0.36	0.0001	0.57	0.0001
	SNCV	0.09	0.28	0.07	0.35	0.15	0.07

SNAPA: sensory nerve action potential amplitude; DSL: distal sensory latencies; SNCV: sensory nerve conduction velocities; HRV DB: heart rate variability deep breathing; HRV OC: heart rate variability orthostatic changes; *r*—coefficient of correlation).

**Table 5:** Results of correlations between the electrophysiological parameters of both motor and sensory nerve function and clinical and demographic selected data.

Nerve			Age		Diabetes Duration		HgbA1c	
			*r*-Values	*p*-Values	*r*-Values	*p*-Values	*r*-Values	*p*-Values
Motor	Median	CMAPA	−0.12	0.13	−0.51	<0.0001	−0.22	0.006
		MNCV	−0.11	0.15	−0.25	<0.0001	−0.13	0.10
	Ulnar	CMAPA	−0.10	0.20	−0.53	<0.0001	−0.19	0.01
		MNCV	−0.04	0.55	−0.44	<0.0001	−0.31	<0.0001
	Peroneal	CMAPA	−0.19	0.01	−0.61	<0.0001	−0.20	0.01
		MNCV	−0.09	0.25	−0.38	<0.0001	−0.22	0.006
	Tibial	CMAPA	−0.19	0.01	−0.55	<0.0001	−0.24	0.003
		MNCV	−0.03	0.63	−0.48	<0.0001	−0.42	<0.0001
Sensory	Median	SNAPA	−0.10	0.19	−0.59	<0.0001	−0.31	<0.0001
		SNCV	−0.17	0.02	−0.45	<0.0001	−0.18	0.020
	Ulnar	SNAPA	−0.09	0.25	−0.60	<0.0001	−0.35	<0.0001
		SNCV	−0.16	0.04	−0.45	<0.0001	−0.15	0.05
	Sural	SNAPA	−0.02	0.81	−0.61	<0.0001	−0.37	<0.0001
		SNCV	−0.02	0.74	−0.16	0.04	−0.17	0.035

CMAPA: compound muscle action potential amplitude; MNCV: motor nerve conduction velocities; SNAPA: sensory nerve action potential amplitude; SNCV: sensory nerve conduction velocities.

**Table 6:** Multivariate analysis between HRV Valsalva and independent variables.

Variable	Standardised Coefficient	*p* Value (CI = 95%)
	Beta (**β**)	
Median CMAPA	0.38	0.01
Median MNCV	−0.11	0.20
Ulnar CMAPA	0.30	0.02
Ulnar MNCV	0.11	0.23
Peroneal DML	−0.16	0.07
Peroneal CMAPA	−0.06	0.68
Peroneal MNCV	−0.02	0.76
Tibial CMAPA	0.01	0.44
Tibial MNCV	0.29	0.01
Tibial DML	−0.03	0.73
Median SNAPA	−0.15	0.41
Median DSL	0.07	0.43
Ulnar SNAPA	0.33	0.09
Sural SNAPA	0.23	0.04

DML: distal motor latencies; CMAPA: compound muscle action potential amplitude; MNCV: motor nerve conduction velocities; SNAPA: sensory nerve action potential amplitude; DSL: distal sensory latencies; SNCV: sensory nerve conduction velocities; HRV DB: heart rate variability deep breathing).

**Table 7:** Multivariate analysis between HRV DB (dependent variable) and independent variables.

Variable	Standardised Coefficient	*p* Value (CI = 95%)
	Beta (**β**)	
Median DML	0.07	0.39
Median CMAPA	0.13	0.35
Median MNCV	−0.002	0.97
Ulnar CMAPA	−0.13	0.27
Ulnar MNCV	0.07	0.41
Peroneal DML	0.03	0.72
Peroneal CMAPA	0.62	0.01
Peroneal MNCV	0.29	0.01
Tibial CMAPA	−0.06	0.62
Tibial MNCV	−0.22	0.05
Median SNAPA	0.25	0.12
Median SNCV	0.16	0.03
Ulnar SNAPA	−0.29	0.08
Ulnar SNCV	−0.09	0.23
Sural SNAPA	0.66	0.0001

DML: distal motor latencies; CMAPA: compound muscle action potential amplitude; MNCV: motor nerve conduction velocities; SNAPA: sensory nerve action potential amplitude; SNCV: sensory nerve conduction velocities; HRV DB: heart rate variability deep breathing.
